# The Continuous Concentration of Particles and Cancer Cell Line Using Cell Margination in a Groove-Based Channel

**DOI:** 10.3390/mi8110315

**Published:** 2017-10-25

**Authors:** Sheng Yan, Dan Yuan, Qianbin Zhao, Jun Zhang, Weihua Li

**Affiliations:** 1 School of Mechanical, Materials and Mechatronic Engineering, University of Wollongong, Wollongong, NSW 2522, Australia; dy983@uowmail.edu.au (D.Y.); qz260@uowmail.edu.au (Q.Z.); 2 School of Mechanical Engineering, Nanjing University of Science and Technology, Nanjing 210094, China; junzhang@njust.edu.cn

**Keywords:** microfluidics, groove-based channel, cell margination, cancer cell

## Abstract

In the capillary venules, blood cells auto-separate with red blood cells aggregating near the centre of vessel and the nucleated cells marginating toward the wall of vessel. In this experiment, we used cell margination to help enrich the Jurkat cells via a groove-based channel which provides a vertical expansion-contraction structure, wherein the red blood cells invade the grooves and push the Jurkat cells to the bottom of the channel. The secondary flows induced by the anisotropic grooves bring the Jurkat cells to the right sidewall. Rigid, 13-µm diameter polystyrene particles were spiked into the whole blood to verify the operating principle under various working conditions, and then tests were carried out using Jurkat cells (~15 µm). The performance of this device was quantified by analysing the cell distribution in a transverse direction at the outlet, and then measuring the cell concentration from the corresponding outlets. The results indicate that Jurkat cells were enriched by 22.3-fold with a recovery rate of 83.4%, thus proving that this microfluidic platform provides a gentle and passive way to isolate intact and viable Jurkat cells.

## 1. Introduction

Cancer cells in the peripheral blood can potentially act as biomarkers for prognosis, evaluation of treatment efficacy, and studying molecular alterations under therapy [1,2,3]. Diagnosis is typically made by blood tests because extracting blood from cancer patients is clinically non-invasive. The conventional approach for cancer cell isolation (e.g., centrifugation and cell lysis) involves multiple steps such as pipetting, changing the sample containers, and chemical treatments, which may result in losing the cancer cells and have an adverse effect on the cells of interest [4]. Since microfluidic isolation platforms can handle small quantities of reagents and samples, while also being able to integrate with other components to form a complete system and provide the user with a one-step process in a single microchip (i.e., flow in—result out), their rapid development provides a useful tool for point-of-care diagnosis [5]. 

Microfluidic separation techniques can separate cells based on magnetic susceptibility, dielectric property, size, and deformability [6]. Cancer cells can be effectively separated using dielectrophoretic [7] or magnetophoretic forces [8]. However, the device always requires complicated fabrication (i.e., electrodes) and bulky equipment (e.g., power supply, electromagnets). Hydrodynamics, a passive method, can separate cancer cells in a label-free manner with a simple setup. Separating by size via filtration [9,10,11] and inertial microfluidics [12,13,14,15] is considered to be a powerful and promising technique for enriching cancer cells due to the high recovery yield and throughput. Filtration is currently challenged as the filter microstructures become clogged after processing a lot of cells; furthermore, captured cells are released based on a reverse flow; this means that the throughput of the filtration approach is limited by clogging and having to operate in batches. Di Carlo’s group [16] proposed a size-selective collection of circulating tumor cells (CTCs) using inertial focusing and micro-scale vortices, which can achieve the high-purity extraction of CTCs from blood. Even though inertial microfluidics provide for continuous cancer cell isolation, the intense cell-to-cell interactions of whole blood (~45% hematocrit) definitely cause a disturbance in inertial focusing; this is why blood samples must be well diluted before being introduced into inertial microfluidic chips.

To overcome these restrictions, we propose a continuous concentration of Jurkat cells (an immortalised line of T leukemia cells) from undiluted blood in a groove-based channel, inspired by leukocyte margination [17,18,19]. In a capillary vessel, the deformable red blood cells (RBCs) prefer to migrate to the axial centre, whereas the less deformable white blood cells (WBCs) tend to localise near the vessel wall [20]. This aggregation of RBCs is attributed to Poiseuille flow with a blood vessel where a pressure gradient pushes them towards the centre [21]. During this inward migration of RBCs, the physical collisions between RBCs and WBCs moves the WBCs to the vessel wall in a process called margination [22]. This hemodynamic phenomenon has been used in the microfluidic platform to extract plasma [23], enrich leukocytes [24], and isolate malaria-infected RBCs [18]. However, marginating leukocytes requires a channel with a very small cross-section, because a moderate stress rate can only be achieved in a narrow channel at an extremely low flow rate; a higher flow rate will induce a higher stress rate and lead to platelet activation [25]. We combined cell margination and secondary flow to steadily enrich Jurkat cells from undiluted blood in a groove-based channel where deformable RBCs move upwards, “invade” the grooves, and then “squeeze out” the Jurkat cells towards the bottom of the channel; secondary flow is induced by the grooves, which move the Jurkat cells to one sidewall of the channel.

This work demonstrates how to use this hemodynamic phenomenon and secondary flow to enrich Jurkat cells using a technique that can process untreated blood (~45% hematocrit) in a label-free manner, and eliminate the off-chip preparation of samples via dilution and chemical modification; the intact Jurkat cells collected from this chip can then be used for downstream detection techniques such as immunostaining. Since this microfluidic chip does not need a power supply, optical fibre, or magnet, its operational simplicity enables Jurkat cells to be enriched in medical laboratories where a syringe pump is accessible.

## 2. Experimental Details

### 2.1. Methodology

**Figure 1:**
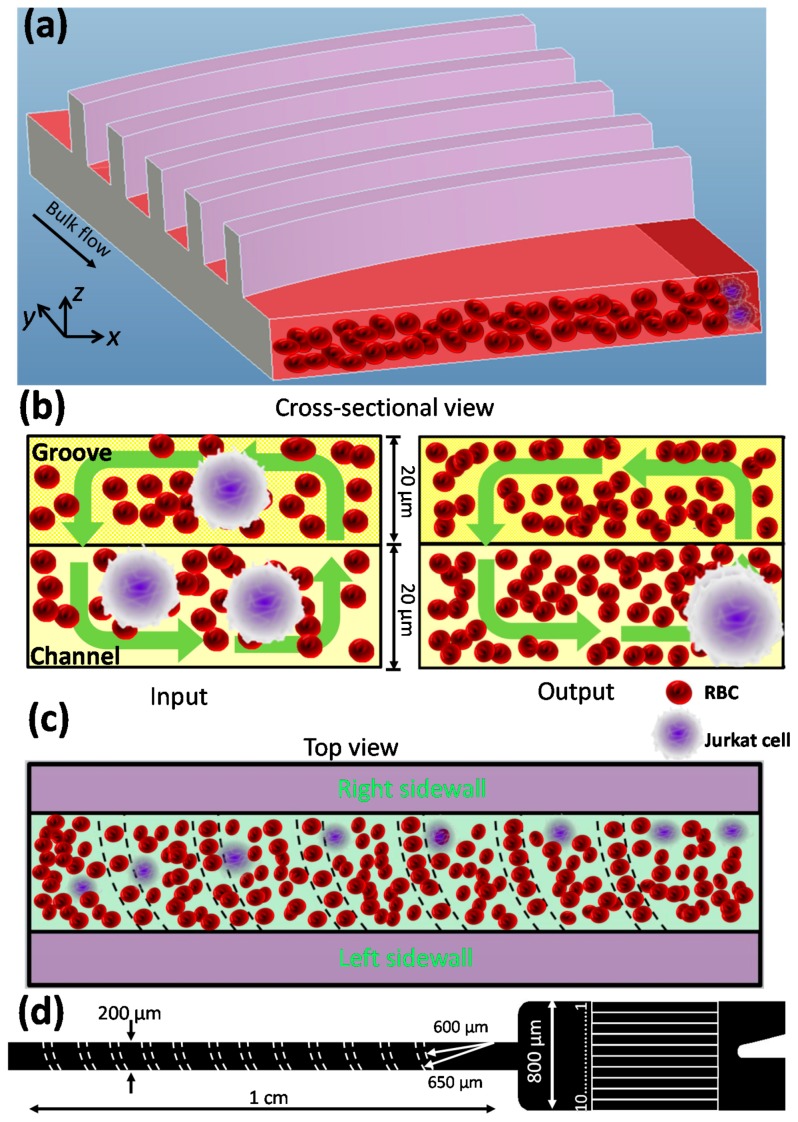
A microfluidic device for Jurkat cell concentration. (**a**) Schematic showing its structure and the spatial distribution of particles. Schematic of the cross-section; (**b**) the top view; and (**c**) the microchannel showing the separation process. The Jurkat cells were distributed randomly at the inlet and migrated to the right sidewall due to a combination of cell margination and secondary flows. (**d**) Optical micrograph images of the groove-based channel.

A schematic of the device is shown in Figure 1a. It consists of an array of grooves where fluid inside the channel is altered by the anisotropic grooves, and secondary flows are induced in the cross-section (Figure 1b); these grooves also provide for vertical expansion and contraction, so when RBCs move upwards and “invade” the grooves, a homotypic adhesion is activated dynamically as the shear stresses decrease in the expansion section, thus forcing the Jurkat cells to move downwards and out of the grooves; the Jurkat cells are then driven to the right sidewall of the channel by secondary flow induced by the anisotropic grooves (Figure 1b,c and Figure S1), and then filtered using a two-outlet system, thus achieving a continuous concentration of Jurkat cells from a sample of undiluted blood. 

### 2.2. Design and Fabrication

Figure 1d shows a schematic of the 200-µm wide by 1-cm long channel where each channel has 60 grooves with a small curvature of 600 μm and a large curvature of 650 μm (Figure 1d and Figure S2). The heights of channel and grooves are both same at 20 μm. The output of the channel was connected to an expansion region (800 μm wide, 20 μm high, and 1 mm long) for better observation and to facilitate data analysis. To study how channel height affects particle migration, a 40-μm deep channel was designed. These microfluidic devices were fabricated by two-step photolithography and soft lithography [26]. Briefly, a mixture of Sylgard 184 elastomer base and curing agent (Dow Corning Corporation, Midland, MI, USA) was cast against the master mold. The cured polydimethylsiloxane (PDMS) replica was peeled off from the mold. The vertical holes for inlet and outlet ports were punched by a blunt needle. Finally, both the PDMS replica and a glass slide were then treated using a plasma cleaner (PDc-002, Harrick Plasma, Ossining, NY, USA) for 3 min, before they were brought into conformal contact for permanent bonding.

### 2.3. Sample Preparation

The sample of human blood extracted from a healthy male volunteer was collected into 10-mL ethylenediaminetetraacetic acid (EDTA) tubes. The blood sample was used immediately after extracting from the volunteer. The Hct of the original blood sample was 45%. To obtain the 1% Hct blood, we used 1× phosphate-buffered saline (PBS, Thermo Fisher, Waltham, MA, USA) to dilute the original blood. Fluorescent particles 13 µm in diameter (Thermo Fisher) were used and particle powder was prepared in the undiluted blood at a final concentration of 5 × 10^5^ particles·mL^−1^. 

### 2.4. Jurkat Cells

The Jurkat cell line was cultured in roswell park memorial institute (RPMI) medium 1640 (Gibco, Sigma-Aldrich, St. Louis, MO, USA) supplemented with 100 units·mL^−1^ of aqueous penicillin, 100 μg·mL^−1^ of streptomycin and 10% of fetal bovine serum at 37 °C in an atmosphere of 100% humidity and 5% CO_2_. The average diameter of Jurkat cells was 15 μm (*n* = 100); they were stained with Calcein AM (Thermo Fisher) for visualisation and quantification, strained and centrifuged at 1000 rpm for 3 min, and then resuspended into the whole blood at a final concentration of 2 × 10^5^ cells·mL^−1^. After passing the microfluidic channel, the viability of Jurkat cells were further verified by the Trypan Blue solution (Thermo Fisher).

### 2.5. Device Characterisation

Prior to the experiments, the chips were sterilised through exposure to UV light for 20 min and then rinsed with 1% bovine serum albumin (BSA) solution (Sigma-Aldrich) to avoid nonspecific adsorption. The spiked blood was fed into the microfluidic chip by syringe pumps (Legato 100, Kd Scientific, Holliston, MA, USA) via Teflon tubes, and then the microfluidic chip was placed onto an inverted microscope (Olympus, Tokyo, Japan). The images were captured by a charge coupled device (CCD) camera (Q-imaging, Albion, Australia) and then post-processed and analysed with Q-Capture Pro 7 (Q-imaging) software. 

To quantify its performance, the distribution of fluorescent particles and stained Jurkat cells was measured from the consecutive images taken at the expansion region. This region was divided into 10 equal bins with a width of 80 µm (Figure 1d) [27] and the distribution of particles and Jurkat cells was defined as the number of particles/Jurkat cells passing through each bin. A custom algorithm was written in the MATLAB software (R2016a, Mathworks, Sydney, Australia), which can convert the images to binary images. Since the image was taken under the fluorescent field, the fluorescent particles or stained cells have large contrast with the background. The software can identify the fluorescent trajectories of beads/cells and measure the number of beads/cells that appeared in each bin. More than 500 beads/cells were counted for each working condition. Two important factors were used to evaluate filtration efficiency with regard to its yield and enrichment [17]. The yield of Jurkat cells was defined as the percentage of total Jurkat cells that collected into the collection outlet, while cell enrichment was defined as the ratio of the purity of Jurkat cells from the collection outlet to the Jurkat cells from the original sample [28]. The cell concentration was analysed using a hemocytometer. Jurkat cell purity was defined as the ratio of the number of Jurkat cells to the total number of cells from the corresponding collection.

## 3. Results and Discussion

### 3.1. Validation of Jurkat Cell Movement

**Figure 2:**
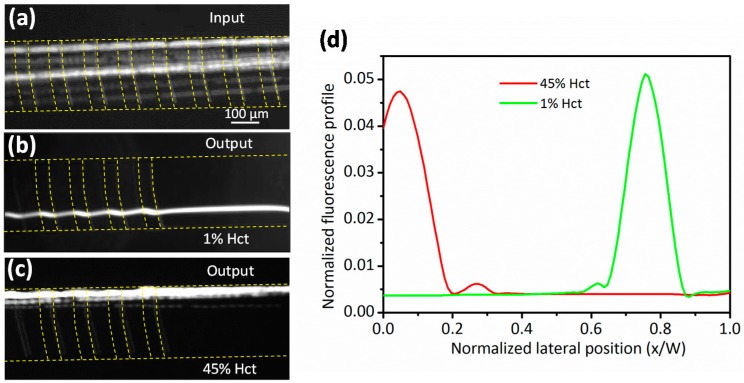
Experimentally focused patterns of 13-µm diameter particles at various sample hematocrits. The applied flow rate was 10 µL·min^−1^. (**a**) Beads were introduced evenly at the inlet. The microscopy images show the trajectories of particles along the hydrophoretic channel at 1% Hct (**b**) and 45% Hct (**c**); (**d**) measured fluorescence profiles of particle trajectories.

To verify the migration of Jurkat cells in highly concentrated blood, rigid 13-µm diameter polystyrene particles were spiked in the blood with 1% and 45% Hct, respectively. All the samples were injected into the groove-based channel at a fixed flow rate of 10 µL·min^−1^. The 13-µm diameter particles were distributed uniformly across the width of the channel at the inlet (Figure 2a). At 1% Hct, all the beads were aligned close to the left sidewall of the channel (Figure 2b). This result is attributed to reduced RBC cell-to-cell interactions and the domination of particle movements by hydrophoresis [29]. Hydrophoresis exploits a steric hindrance mechanism to separate particles under a pressure gradient induced by grooves [30]. Those particles whose diameter is larger than half of the channel height will be dominated by steric hindrance to form hydrophoretic ordering. As Figure 2b shows, the particles in the groove-based channel had helical movements which fluctuated when they passed the grooves, but at a high hematocrit (45% Hct), the beads were displaced to the right sidewall of the channel (Figure 2c). Since the RBCs tended to occupy the grooves, their migration kept the particles out of the grooves, so the rigid particles were followed by secondary flow and focused onto the right sidewall. Since the beads were pushed to the bottom of the channel, no “fluctuated” movements were observed during the passage of the grooves. Without the grooves, the particles will not migrate to the right ridewall (Figure S3).

Figure 2d shows the fluorescent profiles of particle trajectories in different hematocrits. The fluorescence profile of particle trajectories was obtained using the software of Q-capture. Normally, over 200 measurements were taken to obtain one curve. Unlike the particles suspended in the 1% Hct which focused near the left sidewall of the channel, those in 45% Hct marginated to the right sidewall, which simplified the outlet design. Therefore, the existence of a high concentration of RBCs is an important factor to affect particle migration. Moreover, the throughput of the proposed method (~45 million cells/min) is 45 times greater than when using hydrophoretic filtration, thus significantly reducing processing time when processing the same volumes of raw blood.

### 3.2. Effect of Flow Rate

**Figure 3:**
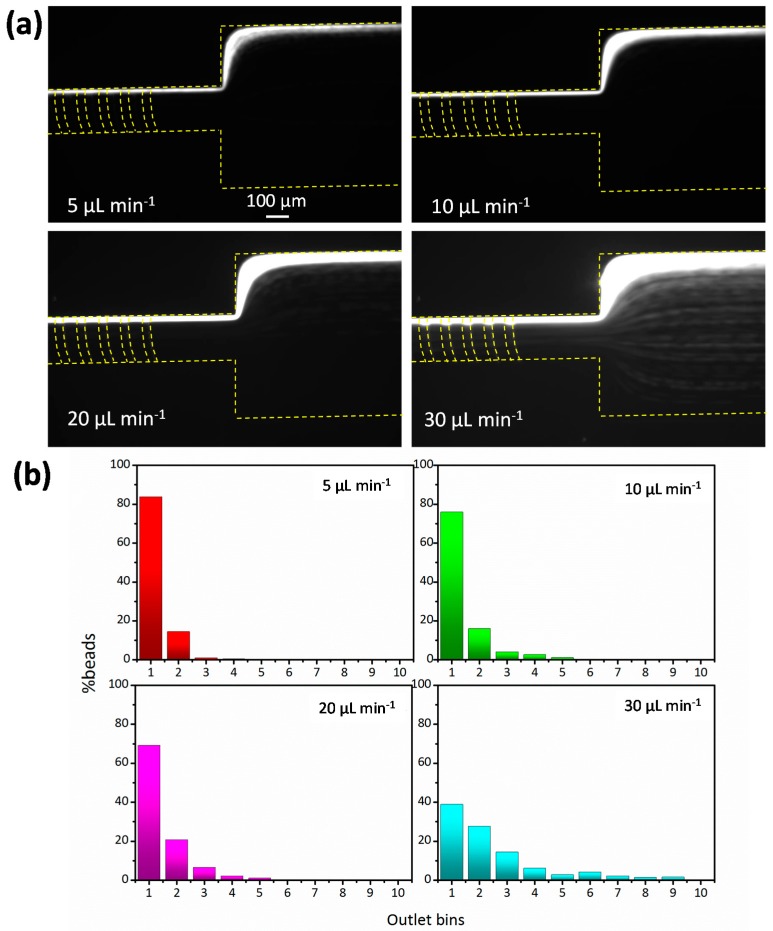
(**a**) Optical micrographs showing particle trajectories at the channel outlet at flow rates of 5, 10, 20, and 30 μL·min^−1^. Particles with a diameter of 13 µm were spiked into the undiluted blood; (**b**) a plot of particle distribution at the channel outlet for varying flow rates. More than 500 beads were counted for each experiment.

After validating the margination phenomenon at 45% Hct in the groove-based channel, experiments were then carried out to determine how the flow rate affected margination efficiency. The results in Figure 3a indicate that a 45% Hct of blood spiked with 13-µm diameter fluorescent particles were pumped through the device at flow rates ranging from 5 µL·min^−1^ to 30 µL·min^−1^. The Reynolds number varied from 0.23 to 1.39. Focusing performance was quantified by counting the number of beads that passed through each bin, of which more than 500 beads were counted for each experiment. Figure 3 shows the distribution of 13-µm diameter particles under various flow rates; qualitatively, the 13-µm diameter particles suspended in undiluted blood focused onto the right sidewall of the channel very well at flow rates of 5 µL·min^−1^, 10 µL·min^−1^, and 20 µL·min^−1^ (Figure 3a). However, although most of the particles remained at the right sidewall, some of them had diffused out of their equilibrium position, possibly because larger drag forces are generated in the cross-section at a higher flow rate (30 µL·min^−1^), which drives the particles into the grooves. Once the particles enter the grooves they will follow the secondary flow and have a “zigzag” motion [31], which results in a wide distribution in the expansion region.

The distribution of beads across the width of the channel is presented quantitatively in Figure 3b. There is a peak distribution in bin 1 for each working condition, and this dropped from 83.8% to 38.9% as the flow rate increased from 5 µL·min^−1^ to 30 µL·min^−1^. When the flow rate was less than 20 µL·min^−1^, more than 90% of the particles passed through bins 1 and 2, a result which suggests that the optimal flow condition for rigid particle focusing should be no more than 20 µL·min^−1^; this optimal flow rate induces a physiological stress (shear stress) that prevents platelet activation. The wall shear rate is 1.65 × 10^3^ s^−1^ at a flow rate of 20 µL·min^−1^, and since the critical stress for platelet activation is about 1000 dyn·cm^−2^ under a given exposure time of 10 s [32], the shear stress of 37.5 dyn·cm^−2^ and retention time of <1 s are safe for platelets to migrate in the channel. Therefore, we did not see any platelet aggregation in the experiments. 

### 3.3. Effect of Channel Height

**Figure 4:**
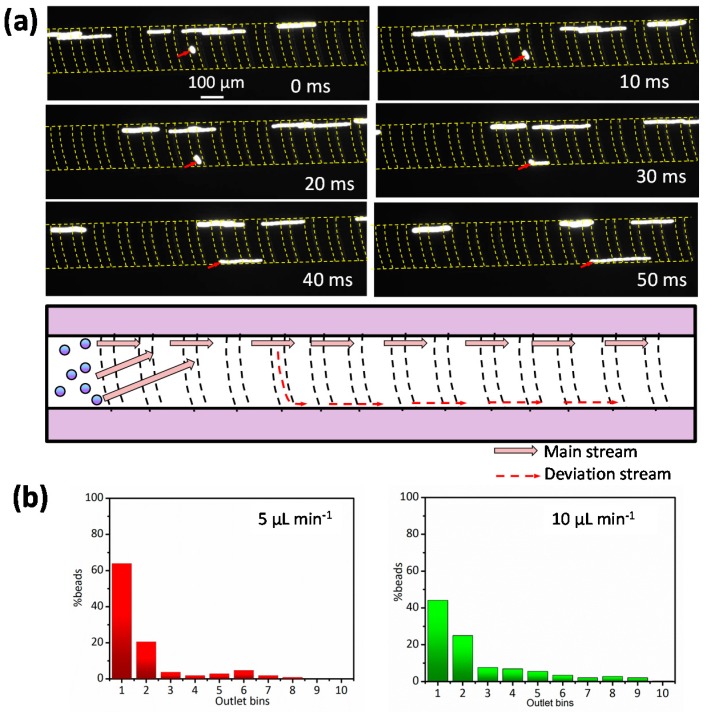
(**a**) The deviation of a particle in a 40-µm high channel showed that some concentrated particles diffused out of their equilibrium position. The images show the top view of the channel; (**b**) a plot of particle distribution at the channel outlet due to the effect of flow rate.

Cell margination is related to the channel geometry. Therefore, we investigated particle migration in a channel with a height of 45 µm; here, 13-µm diameter particles were suspended in 45% Hct blood and then injected into the channel. Figure 4a shows that the particles deviated (as indicated by the red arrow) at a flow rate of 5 µL·min^−1^. To record the clear particle trajectories under a fluorescent field, the exposure time was set at 100 ms and images were taken at intervals of 10 ms. Within 20 ms, the particles in the groove moved to the left sidewall of the channel, following the angle of the groove. Compared to other particles, the highlighted particles had a shorter trajectory under the same exposure time because their velocity in the groove was quite low. This also indirectly demonstrated that the particles migrated in the groove where the fluid velocity was lower than in the main channel (Figure S1), but after 30 ms they reached the left sidewall and then entered the main channel and began to move faster. 

Particle distributions at the outlet bins under the effects of flow rate are plotted in Figure 4b. Unlike the particles in the 20-µm high channel, they were not focused very well. The peak distributions in bin 1 at flow rates of 5 µL·min^−1^ (Re = 0.42) and 10 µL·min^−1^ (Re = 0.84) were 63.8% and 44.1%, respectively, a result that could be attributed to the weak cell-to-cell interaction in a wider space. Some of the concentrated particles entered the grooves, resulting in a process of deviation, as shown in Figure 4a. Therefore, channel height is a key parameter that affects particle migration in undiluted blood.

### 3.4. Jurkat Cell Concentration

**Figure 5:**
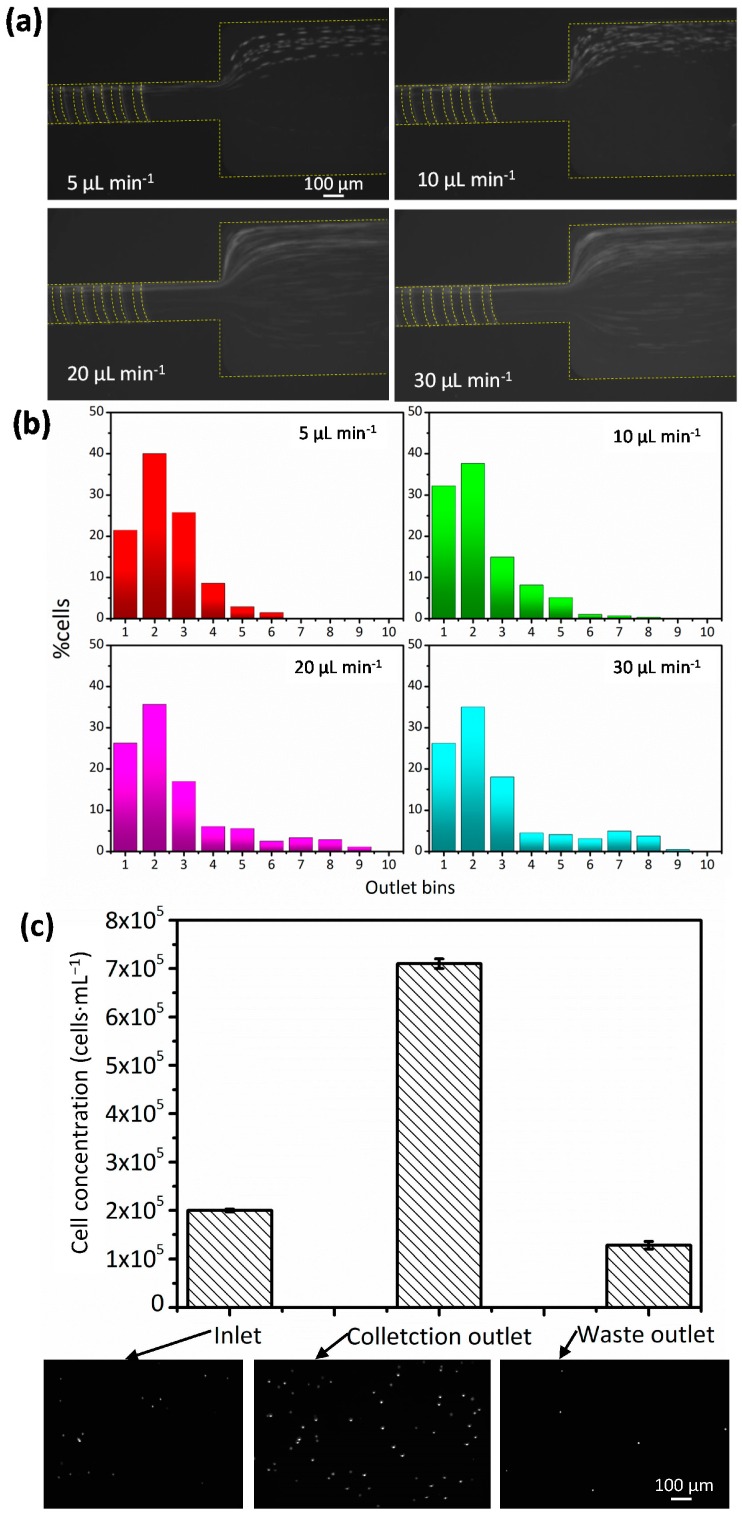
(**a**) Optical micrographs showing cell trajectories at the channel outlet at flow rates of 5, 10, 20, and 30 μL·min^−1^. The Jurkat cells were spiked into undiluted blood; (**b**) a plot of cell distribution at the channel outlet for varying flow rates where more than 500 cells were counted for each experiment; (**c**) the plot showing the concentration of cells collected from the inlet, collection outlet, and waste outlet. The average value was three times the measurement, and the error bar represents standard deviation. The fluorescence images show the Jurkat cells collected from the inlet, collection outlet, and waste outlet; these images were captured at 50× magnification.

After demonstrating the working principle with experiments using 13-µm diameter polystyrene particles, tests with Jurkat cells were then carried out by spiking them into undiluted blood (~45% Hct) to model the presence of Jurkat cells in human peripheral blood. This spiked blood was then pumped through a 20-µm high channel at flow rates ranging from 5 µL·min^−1^ to 30 µL·min^−1^. Figure 5a,b show the trajectories and distributions of Jurkat cells under various flow rates. The Jurkat cells were not tightly focused onto the right sidewall of the channel like the rigid polystyrene particles. A peak distribution was seen in bin 2 rather than bin 1 (closest to the right sidewall), and the optimal flow rate for focusing Jurkat cells decreased to 10 µL·min^−1^. This suggests that Jurkat cells are not as stiff as the hard polystyrene particles and the difference in deformability between Jurkat cells and RBCs is not enough to focus the Jurkat cells onto the right sidewall of the channel. In some clinical settings, the stiffness of cancer cells may be unknown or vary. However, the cancer cells are nucleated and the stiffness of nucleated cells is about two orders of magnitude greater than RBCs [33]. Therefore, even the stiffness of cancer cells vary, and cell margination can still happen in this device.

Since these experiments were carried out to investigate how the flow rate affected cell focusing, separated Jurkat cells were collected to calculate how efficient filtration was at an optimal flow rate of 10 µL·min^−1^. Figure 5c shows the concentration of cells collected from the inlet, the collection outlet, and the waste outlet. The images were taken in a hemocytometer under the fluorescent field. The enrichment ratio of Jurkat cells in the collection outlet was 22.3 ± 2.1, and the recovery rate for this filtration process was 83.4% ± 2.3%. However, enrichment can be improved even further by a multi-stage process or by optimising the bifurcated outlets. Since viable Jurkat cells play very significant roles in downstream applications, including gene expression [34] and drug delivery [35], their viability was tested to verify the usefulness of the proposed device. Up to 99.2% cells remained viable after passing the channel, which proved that this device was a gentle and non-invasive way for Jurkat cell concentration. 

This technique uses expansion-constriction structures that induce secondary flows and cell margination for Jurkat cell concentration, a process with many distinct advantages. First, the device operates at a continuous mode, enabling a high throughput (~10 µL·min^−1^, 4.5 × 10^7^ cells/min), and the cells migrate at an optimal flow rate where Re = 0.15, which is similar to arteriole flow [36], and provides a physiological environment where platelets will not be activated. Moreover, since this device can process whole blood directly extracted from patients, preliminary steps such as pre-dilution and RBC lysis are not required [17], further saving processing time and cost. Also, by eliminating the bulk field generator, this passive microfluidic chip is portable for outfield settings.

## 4. Conclusions

This work proposed a groove-based channel for the continuous isolation of Jurkat cells from undiluted blood. Here, less-deformable cancer cells (Jurkat) were forced to the bottom of the channel after passing a series of grooves and were then brought to the right sidewall by secondary flows. Rigid, 13-µm diameter polystyrene particles were spiked into the undiluted blood and used to verify particle migration in the channel. Due to the deformability and transverse flows of RBCs, the particles migrated rapidly to the right sidewall of the channel. More than 90% of the particles passed through bins 1 and 2 at a flow rate of less than 20 µL·min^−1^. A similar result was seen as Jurkat cells migrated in whole blood, but the Jurkat cells were not concentrated as much because they were not as stiff as the polystyrene beads. After filtration, Jurkat cells were enriched 22.3-fold ± 2.1-fold with a recovery rate of 83.4% ± 2.3%. Since this passive microfluidic chip can directly process whole blood, thus eliminating sample preparation and complex external force fields, it can potentially be miniaturised and used as portable equipment for detecting Jurkat cells. 

However, the throughput of this device is still limited. To significantly enhance the throughput, parallelised channels should be designed, where multiple channels can operate simultaneously for blood processing. Compared with label-based cell enrichment, this label-free method can reduce the time of sample handling and eliminate the need for antibodies. Besides, this device can directly process whole blood without any preliminary steps, further saving processing cost and time.

## Supplementary Materials

The following are available online at www.mdpi.com/2072-666X/8/11/315/s1, Figure S1: (a) Schematic diagram of a 2D model; (b) the simulated results of the flow field, pressure, and shear rate. The anisotropic microstructure induces secondary flows. The arrows are velocity vectors; (c) the simulated results of flow field as seen from the side, Figure S2: (a) Mask design of groove-based channel; (b) optical image showing the structure of the groove-based channel, Figure S3: Measured fluorescence profiles of particle distribution at the outlet of the channel with grooves and without grooves. Without grooves, the particles were evenly distributed at the output. With the grooves, the particles were concentrated along the channel.
