# Supplementary material for: The Continuous Concentration of Particles and Cancer Cell Line Using Cell Margination in a Groove-Based Channel
Source: Micromachines (Basel). 2017 Oct 25;8(11):315. doi: 10.3390/mi8110315 (PMC6189968; doi:10.3390/mi8110315)
Supplement: Supplementary file 1 [file micromachines-08-00315-s001.pdf]

micromachines

Supplementary Materials: The continuous concentration of particles and cancer cell line using cell margination in a groove-based channel

Sheng Yan, Dan Yuan, Qianbin Zhao, Jun Zhang and Weihua Li

Computational model

The flow characteristics of the channel were studied numerically using the finite element software (COMSOL Multi-physics 5.0 COMSOL, Burlington, MA, USA). A 3D model was built with geometry that is identical to the experimental channel. The laminar flow module was utilized to solve fluid flow in the three-dimensional model; a no-slip boundary condition was set to the channel walls; the blood was assumed to be an incompressible flow with a density of 1.06×10^3 kg·m^{-3} and a constant viscosity of 4 cP, and the flow rate at the inlet was set at 10 μL·min^{-1} and the pressure at the outlet was set at zero. The pressure and velocity vectors on the cross sections can be solved in the "Navier–Stokes Mode". The groove-based channel was meshed using Free Triangular with 652,113 elements, and a stationary solver was used to compute the flow field (Figure S1).

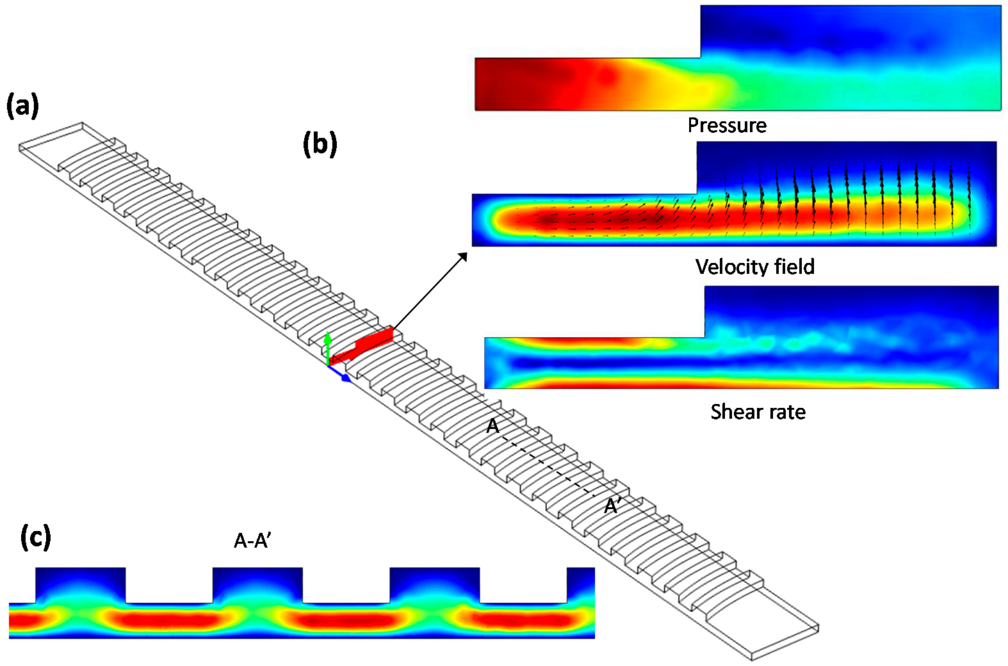

Figure S1. (**a**) Schematic diagram of a 2D model; (**b**) the simulated results of the flow field, pressure, and shear rate. The anisotropic microstructure induces secondary flows. The arrows are velocity vectors; (**c**) the simulated results of flow field as seen from the side.

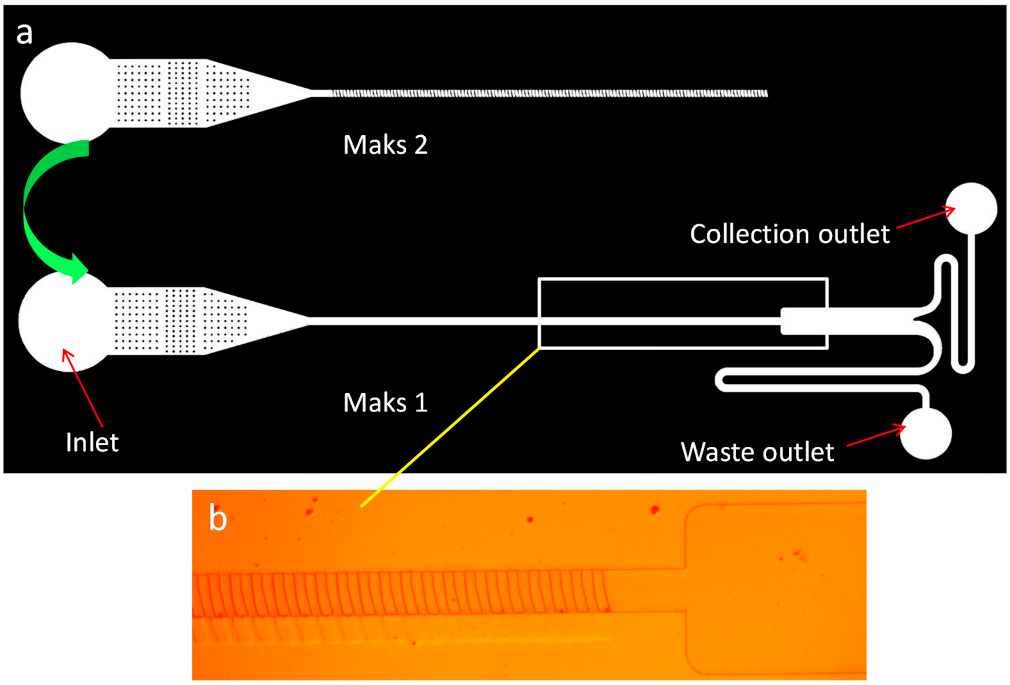

Figure S2. (**a**) Mask design of groove-based channel; (**b**) optical image showing the structure of the groove-based channel.

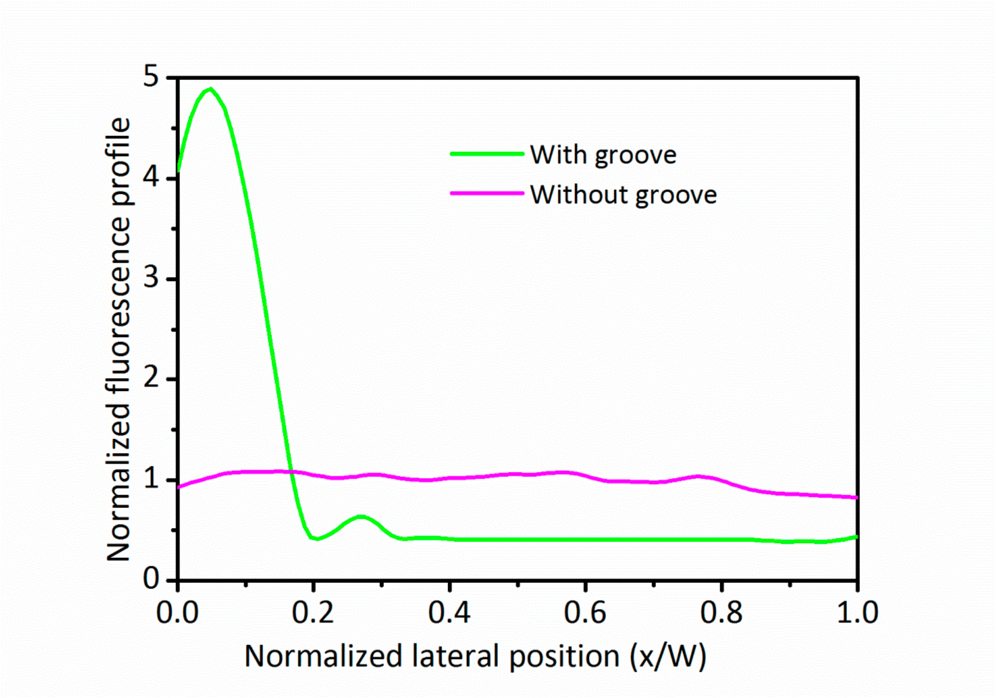

Figure S3. Measured fluorescence profiles of particle distribution at the outlet of the channel with grooves and without grooves. Without grooves, the particles were evenly distributed at the output. With the grooves, the particles were concentrated along the channel.
